# Health, wellbeing, and disability among older people infected or affected by HIV in Uganda and South Africa

**DOI:** 10.3402/gha.v6i0.19201

**Published:** 2013-01-23

**Authors:** Makandwe Nyirenda, Marie-Louise Newell, Joseph Mugisha, Portia C. Mutevedzi, Janet Seeley, Francien Scholten, Paul Kowal

**Affiliations:** 1 Africa Centre for Health and Population Studies, University of KwaZulu-Natal, Somkhele, South Africa; 2 School of Social Sciences, University of Southampton, Highfield, Southampton, UK; 3 Centre for Paediatric Epidemiology and Biostatistics, UCL Institute of Child Health, London, UK; 4 Medical Research Council/Uganda Research Unit on AIDS, Uganda Virus Research Institute, Entebbe, Uganda; 5 London School of Hygiene and Tropical Medicine, London, UK; 6 School of International Development, University of East Anglia, Norwich, UK; 7 Multi-Country Studies Unit, World Health Organization, Geneva, Switzerland; 8 Research Centre on Gender, Health and Ageing, University of Newcastle, Newcastle, Australia

**Keywords:** South Africa, Uganda, older people, health status, functional ability, subjective wellbeing

## Abstract

**Objective:**

To describe and compare the health status, emotional wellbeing, and functional status of older people in Uganda and South Africa who are HIV infected or affected by HIV in their families.

**Methods:**

Data came from the general population cohort and Entebbe cohort of the Medical Research Council/Uganda Virus Research Institute, and from the Africa Centre Demographic Information System through cross-sectional surveys in 2009/10 using instruments adapted from the World Health Organization (WHO) Study on Global Ageing and adult health (SAGE). Analysis was based on 932 people aged 50 years or older (510 Uganda, 422 South Africa).

**Results:**

Participants in South Africa were slightly younger (median age − 60 years in South Africa, 63 in Uganda), and more were currently married, had no formal education, were not working, and were residing in a rural area. Adjusting for socio-demographic factors, older people in South Africa were significantly less likely to have good functional ability [adjusted odds ratio (aOR) 0.72, 95% CI 0.53–0.98] than those in Uganda, but were more likely to be in good subjective wellbeing (aOR 2.15, 95% CI 1.60–2.90). South Africans were more likely to be obese (aOR 5.26, 95% CI 3.46–8.00) or to be diagnosed with hypertension (aOR 2.77, 95% CI 2.06–3.73).

**Discussion and conclusions:**

While older people’s health problems are similar in the two countries, marked socio-demographic differences influence the extent to which older people are affected by poorer health. It is therefore imperative when designing policies to improve the health and wellbeing of older people in sub-Saharan Africa that the region is not treated as a homogenous entity.

In sub-Saharan Africa and other developing countries, there is limited information on the general health and wellbeing of persons aged 50 years and above, and even less is available on the topic of older adults and HIV (1–5). HIV remains a major public health challenge in sub-Saharan Africa, with Uganda and South Africa among the worst affected. According to the 2011 Uganda AIDS Indicator Survey, an estimated 6.7% of the population in Uganda was HIV infected (6). South Africa on the other hand, with an estimated adult HIV prevalence rate of 16.2%, has the world’s highest number of people living with HIV: around 5.6 million (7), with nearly 2 million on antiretroviral treatment (ART). It has been suggested that there may have been a decline in annual HIV incidence rates in certain age groups between 2001 and 2009 (8). Studies on people aged 50 and above in Uganda and South Africa estimate the HIV incidence rates in these populations at 0.2 and 0.5%, respectively (9, 10). Furthermore, although incidence rates are low, an increasing number of older people are living with HIV as a result of improved survival on ART (11–14). However, although HIV is a recognised public health problem in both Uganda and South Africa, neither country has much data on the effects of HIV on the health and wellbeing among older people infected or affected by HIV (15).

Studies from Kenya (16), Tanzania (17), Ghana (18), and South Africa (19, 20) have provided recent empirical evidence on the health status of older people. A shortcoming of these studies is that HIV status was not assessed, whereas other studies exclusively focused on HIV-infected older persons (21, 22). Other researches on the health status of older people in sub-Saharan Africa have thus far largely been based on qualitative studies (23) or have utilised a limited set of health domains (24).

This article is a follow-up of separate analyses conducted in Uganda and South Africa (25, 26), using survey data collected in 2009/10 with standardised World Health Organization (WHO) questionnaires and analytical instruments (27). Previous findings from these analyses showed age to be a key determinant of health and functional ability in both countries, with men reporting better health and functional status than women (25, 26). In Uganda, apart from lower body mass index (BMI), HIV-infected older adults on ART reported similar health and functional ability as HIV-uninfected participants (25). In South Africa, HIV-infected and HIV-uninfected women were significantly less likely than men to report very good or good health. HIV-infected South Africans had better functional ability, quality of life, and overall health status than HIV-uninfected participants (26).

In the analysis presented here, data from the two studies have been pooled for a systematic, comparative analysis of the health and wellbeing of older people. The aim of this article is to describe and compare self-rated health, subjective wellbeing and functioning of older people in Uganda and South Africa who are HIV infected or affected by HIV in their families.

## Methods

### Study setting

Data used in this analysis were collected from the general population cohort and Entebbe cohort of the Medical Research Council/Uganda Virus Research Institute (MRC/UVRI) in Uganda (25), and from the Africa Centre surveillance area in rural South Africa (26) through cross-sectional surveys in 2009/10. The general population cohort (GPC) is located in rural Kalungu district, southwestern Uganda, established by the MRC/UVRI in 1989. The GPC collects demographic and behavioural information, such as births, deaths, access to health care, and sexual partnerships in annual surveillance rounds. The Entebbe cohort was established by MRC/UVRI in 1994 to conduct epidemiological and clinical studies relating to HIV/AIDS. Further details about the open population cohort and the Entebbe cohort have been previously described (28–30).

The Africa Centre surveillance is located in northern rural KwaZulu-Natal, South Africa. The surveillance area is predominantly rural, only less than 10% of the approximately 90,000 household members under surveillance live in areas defined as urban. Demographic, social, and health information, such as births, deaths, migrations, and health care utilisation, are collected from all consenting households and their members (31) in twice yearly surveillance rounds since 2000. In addition, social and economic information such as household asset ownership, sanitation facilities, energy sources, and access to social amenities is collected in special annual modules of the demographic surveillance. A nested annual sexual behaviour and HIV surveillance has been operating among adults aged 15 years and above since 2003. The adult HIV prevalence in the South African study site has been estimated at around 22% with prevalence by age as high as 50% among women aged 30–34 years (32). The Africa Centre surveillance has been described in detail elsewhere (31, 33) or www.africacentre.com.

### Study population

In both Uganda and South Africa, the study sample was made up of persons aged 50 years and above who resided within the respective surveillance populations. The study randomly selected 100 older persons in each of the five distinct groups based on the surveillance fieldsite census records and nested surveys described above. Refusals were less than 1%. The categorisation into the five groups was based on the following criteria:

1. Older persons who were HIV infected and on ART for a year or longer;
2. Older persons who were HIV infected and not yet on ART or on ART for three or less months;
3. Older persons living with an adult child who was HIV infected and on ART;
4. Older persons who had an adult child who died of HIV-related death; and
5. Older person who was HIV uninfected and had no adult child who was HIV infected.

The fifth group was the comparison group but could only be established in the Ugandan study as in South Africa, with the high HIV prevalence (32), older HIV-uninfected people not affected by HIV in the family were rare.

### Data collection

Data from both sites were collected using structured questionnaires adapted from survey instruments of the WHO’s Study on global AGEing (SAGE) and adult health (27, 34). The health measurement tools included in the survey instruments have been validated and shown to be applicable across different settings (35–38). The tools were translated into respective local languages and modified to take into account the local context. The questionnaires had four main parts: demographic and household information; health and wellbeing assessment; care-giving and care-receiving patterns; and anthropometric measurements. At the Uganda site, in addition to measured weight and height, objective health measures, including blood pressure, hand grip strength, near and distant vision, walking speed, and cognition, were collected. Blood samples were collected at the South African site. In both sites, face-to-face interviews were used to collect data on persons aged 50 years and above, with further details published elsewhere (25, 26).

### Variables

Age, sex, marital status, education, occupation, place of residence, and measures of household wealth were collected. Age reporting was checked against surveillance records. Income quintiles were generated from household ownership of durable goods, dwelling characteristics (type of floors, walls, and cooking stove), and access to services, such as improved water, sanitation, and cooking fuel. An ‘asset ladder’ was generated and using a Bayesian post-estimation (empirical Bayes) method, households were arranged on the asset ladder, where the raw continuous income estimates were transformed into quintiles. In addition, a question about self-perceived financial status was asked, ‘Compared to 3 years ago, would you say your financial situation now is better or worse off?’.

Multiple measures of health and wellbeing were used in this analysis as outcome variables (functional disability, emotional well-being, prevalence of self-reported hypertension, BMI, and self-rated general overall health). The WHO Disability Assessment Schedule (WHODAS) (39) was used to assess the functional ability of study participants. A series of questions were asked on the ability of participants to perform tasks, such as walking, standing, stooping, and climbing a flight of stairs. The 12-item WHODAS instrument (38) was used to obtain a measure of functional ability, with results transformed to a scale of 0–100, where 0 represented poorest functional ability (highest disability) and 100 represented the best functional ability (lowest disability). The science of measuring happiness often includes both experienced wellbeing and evaluative wellbeing. In this study, evaluative wellbeing was measured using the WHO Quality of Life instrument (WHOQOL) (35). The eight-item version of WHOQOL assesses satisfaction with, amongst other things, life, health, and living conditions. The resulting raw score from the eight questions ranged from 8 to 40. This was later transformed into a scale from 0 to 100, where 100 represented best subjective wellbeing.

Measurements of height and weight were taken of all participants. These measurements were used to compute the BMI, using a standard formula of weight in kilograms over height in metres (squared). WHO’s BMI classifications were used where BMI of less than 18.5 is considered under-weight, while between 25.0 and 29.9 are considered over-weight, and 30+ is considered obese (40). BMI is presented as a mean value or, when in the regression model, is collapsed into a dichotomous variable (not obese = BMI < 30 or obese BMI ≥ 30). Prevalence of hypertension was calculated based on respondent self-report to a question, *Have you ever been told by a health care professional that you have high blood pressure/hypertension?* Self-rated health was obtained from a single overall general health question, *In general, how would you rate your health today?* This question has been demonstrated to be a relatively robust indicator of health and mortality across different settings (36, 41), even though it has some consistency and comparability shortcomings. The response categories were based on a 5-point Likert-type scale: ‘very good’, ‘good’, ‘moderate’, ‘bad’, and ‘very bad’. The first three categories were collapsed into a rating of ‘good’ and the last two into ‘bad’.

### Data analysis

Indicators of health, functioning, and wellbeing were examined in bivariate and multivariable analyses. A multivariable logistic regression analysis was done to assess the health and wellbeing status of participants in Uganda relative to those in South Africa. This relationship was assessed adjusting for age, sex, education, occupation status, household wealth quintiles, change in self-perceived household financial status, and rural versus peri-urban place of residency. For these analyses, a dichotomous variable of good versus poor physical health or subjective wellbeing was generated. The categorisation into good or poor physical health was based on falling above or below an overall WHODAS median value of 80.5 for the pooled sample. For subjective wellbeing, an overall WHOQoL median cut-off value of 62.5 was used. Stata statistical software version 11.2 was used for all analyses (42).

Ethics approval was obtained from the Uganda Virus Research Institute, Science and Ethics Committee and the National Council for Science and Technology for the Uganda project, and from the University of KwaZulu-Natal Biomedical Research Ethics committee for the South Africa project.

## Results

A total of 932 participants (510 in Uganda and 422 in South Africa) were included in the final sample. Women made up 75% (South Africa) and 61% (Uganda) of the respective samples (Table 1). Overall, participants in Uganda were slightly older than in South Africa; median age was 63 years in Uganda (66 years men, 62 years women) and 60 years in South Africa, for both men and women. A major difference pertained to employment status where 94% of the sample in South Africa was not working, but 81% of the Uganda participants reported to be working. Other significant differences in the study samples were observed in marital status, education attainment, self-perceived financial status, and place of residency (Table 1).

**Table 1:** Socio-demographic characteristics of study participants by country

	South Africa		Uganda		
	
	*n*	%	*n*	%	*p*
Median age (age range)	60 (50–94)		63 (50–96)		
Sex					<0.001
Male	106	25.1	198	38.8	
Female	316	74.9	312	61.2	
Age group					0.002
50–59	190	45.0	178	34.9	
60–69	128	30.3	150	29.4	
70–79	74	17.5	127	24.9	
80 +	30	7.1	55	10.8	
Marital status					<0.001
Never married	116	27.5	7	1.4	
Married	206	48.8	165	32.4	
Separated/divorced	8	1.9	105	20.6	
Widowed	91	21.6	233	45.7	
Missing	1	0.2	0	0.0	
Education level					<0.001
NFE/AEO	201	47.6	118	23.1	
Primary	165	39.1	293	57.5	
Secondary	52	12.3	71	13.9	
Tertiary	4	0.9	25	4.9	
Missing	0	0.0	3	0.6	
Employment status					<0.001
Working	24	5.7	411	80.6	
Not working	395	93.6	87	17.1	
Missing	3	0.7	12	2.4	
Financial status					<0.001
Better	67	15.9	20	3.9	
About the same	136	32.2	56	11.0	
Much worse	219	51.9	426	83.5	
Missing	0	0.0	8	1.6	
Wealth quintiles					1.000
First (lowest)	85	20.1	102	20.0	
Second	84	19.9	102	20.0	
Third	86	20.4	102	20.0	
Fourth	84	19.9	102	20.0	
Fifth (highest)	83	19.7	102	20.0	
Place of residency					0.042
Peri-urban/urban	182	43.1	254	49.8	
Rural	240	56.9	256	50.2	

Table 2 shows age–sex adjusted physical functional ability, subjective wellbeing, and BMI for older people in South Africa and Uganda. In both countries and across the study groups, men reported better physical functional ability and subjective wellbeing than women. At an aggregate level, age–sex adjusted mean physical functional ability in South Africa was 72.7 (95% CI 71.1–74.4) compared to 73.5 (95% CI 72.0–75.1) in Uganda. Subjective wellbeing scores were higher in South Africa (59.0, 95% CI 57.6–60.4) than Uganda (55.8, 95% CI 54.7–57.0), suggestive of higher satisfaction with their life, health, and living arrangements among study participants in South Africa relative to participants from Uganda. Being obese (BMI ≥ 30) was common among women participants in both sites, and by study group, with the exception being participants who had experienced the death of an adult child due to a HIV-related cause in Uganda. Overall, a higher proportion of participants in South Africa (27.8, 95% CI 27.1–28.5) than Uganda (25.2, 95% CI 24.0–26.5) were found to be obese.

**Table 2:** Age–sex adjusted mean functional ability, subjective wellbeing, and body mass index by study group, sex, and country (95% confidence interval in parenthesis)

Study groups	South Africa			Uganda		
	
	Male	Female	Both sexes	Male	Female	Both sexes
	Functional abilitya^a^			Functional ability		
HIV+, on ART	87.2 (84.7–89.6)	78.2 (74.7–81.7)	81.9 (79.6–84.2)	79.8 (74.4–85.1)	76.9 (72.4–81.4)	77.9 (74.5–81.4)
HIV+, no ART/ART <3 months	77.1 (73.5–80.6)	68.9 (65.7–72.1)	70.8 (68.2–73.4)	79.9 (75.0–84.7)	67.7 (60.4–75.0)	71.7 (66.5–76.8)
Has HIV+ adult child	83.4 (79.9–87.0)	70.0 (66.2–73.7)	73.9 (71.1–76.8)	78.6 (73.8–83.4)	71.0 (67.0–75.0)	73.5 (70.4–76.6)
Has adult child, died with HIV	76.0 (69.5–82.6)	68.3 (64.8–71.8)	70.6 (67.4–73.7)	82.5 (77.3–87.6)	73.1 (69.6–76.6)	76.2 (73.3–79.0)
Comparison^b^	n/a	n/a	n/a	79.1 (74.4–83.8)	72.2 (69.1–75.4)	74.5 (71.9–77.1)
Total	79.6 (76.6–82.5)	69.4 (67.5–71.4)	72.7 (71.1–74.4)	78.6 (76.3–80.9)	71.1 (69.1–73.1)	73.5 (72.0–75.1)
						
	Subjective wellbeing^c^			Subjective wellbeing		
HIV+, on ART	66.4 (62.9–69.8)	60.0 (56.2–63.8)	62.6 (59.9–65.2)	64.6 (60.5–68.7)	59.0 (56.0–62.1)	61.0 (58.5–63.4)
HIV+, no ART/ART <3 months	61.2 (56.9–65.5)	53.6 (48.9–58.4)	55.4 (51.6–59.2)	59.5 (55.8–63.1)	53.2 (47.3–59.2)	55.3 (51.1–59.4)
Has HIV+ adult child	62.6 (60.6–64.5)	59.5 (56.6–62.4)	60.4 (58.3–62.5)	56.75 (1.6–61.7)	53.9 (50.8–56.9)	54.8 (52.2–57.4)
Has adult child, died with HIV	62.0 (55.0–69.0)	57.2 (54.6–59.7)	58.6 (55.9–61.3)	55.3 (48.7–61.9)	54.1 (51.1–57.2)	54.5 (51.6–57.5)
Comparison^b^	n/a	n/a	n/a	57.5 (53.8–61.3)	54.9 (51.5–58.2)	55.6 (53.2–58.3)
Total	62.4 (59.7–65.0)	57.4 (55.7–59.0)	59.0 (57.6–60.4)	57.9 (56.0–59.8)	54.8 (53.4–56.3)	55.8 (54.7–57.0)
						
	Body mass index			Body mass index		
HIV+, on ART	25.3 (23.2–27.4)	26.9 (25.3–28.4)	26.2 (25.0–27.5)	24.9 (19.0–30.9)	24.3 (19.0–29.6)	24.5 (20.5–28.5)
HIV+, no ART/ART <3 months	24.4 (22.9–25.9)	27.5 (25.7–29.2)	26.7 (25.4–28.1)	20.4 (19.6–21.2)	24.6 (21.9–27.3)	23.3 (21.4–25.1)
Has HIV+ adult child	25.7 (24.6–26.8)	30.3 (28.8–31.8)	29.0 (27.9–30.0)	21.1 (19.4–22.9)	26.6 (24.3–28.8)	24.8 (23.2–26.4)
Has adult child, died with HIV	24.8 (22.8–26.9)	31.2 (29.5–32.9)	29.3 (28.0–30.7)	36.3 (22.4–50.3)	26.9 (24.6–29.1)	30.0 (25.2–34.7)
Comparison^b^	n/a	n/a	n/a	24.4 (20.4–28.5)	28.0 (24.4–31.7)	26.9 (24.1–29.6)
Total	24.5 (23.4–25.8)	29.4 (28.6–30.3)	27.8 (27.1–28.5)	23.8 (21.7–25.9)	25.9 (24.3–27.5)	25.2 (24.0–26.5)

a Functional ability, as measured by a WHODAS, with score transformed to 0 to 100, where 0 represents poorest functional ability (highest disability) and 100 best functional ability (lowest disability).

b Study group only established in Uganda, not in South Africa.

c Subjective well-being, as measured by a WHOQoL, with score transformed into a scale from 0 to 100, where 100 represented best subjective wellbeing.

**Fig. 1:**
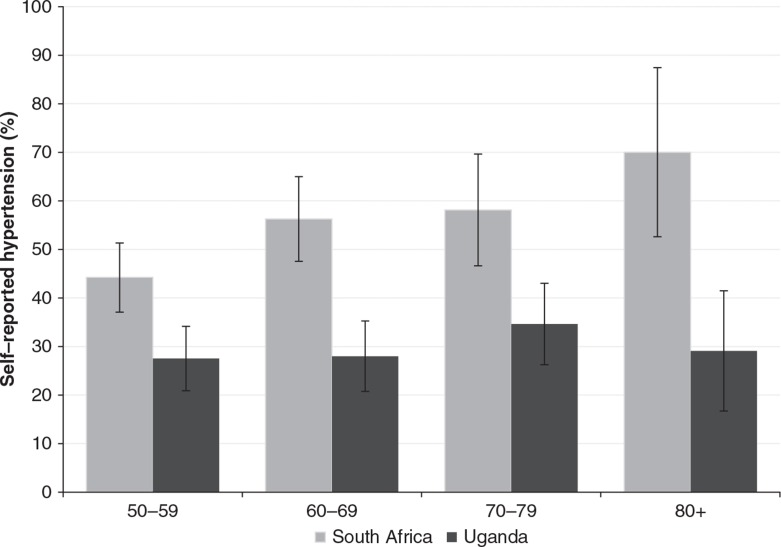
Prevalence of self-reported hypertension by age group with 95% confidence interval, South Africa and Uganda, 2010.

**Fig. 2:**
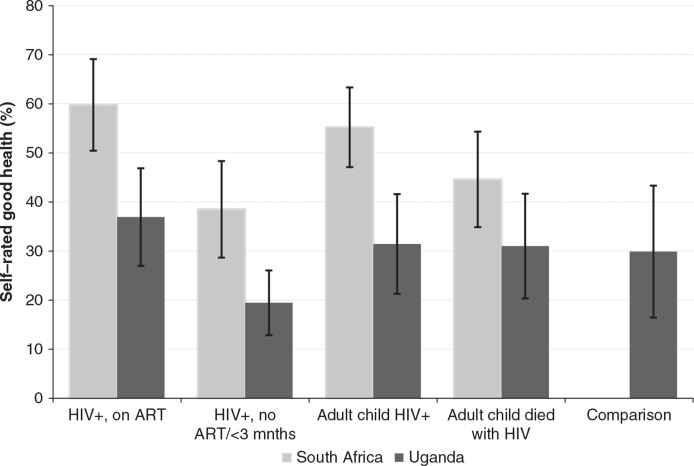
Proportion (mean and 95% CI) of self-reported good health by study group, South Africa and Uganda, 2010.

Participants in South Africa reported a significantly higher prevalence of ever having been diagnosed with hypertension (Fig. 1). In South Africa, the prevalence of ever being diagnosed with hypertension increased with age from about 45% in the 50–59 age group to around 70% among those aged above 80. In Uganda, there was only a modest increase between the 50–59 and the above 80 age groups. When asked how they rated their overall general health, adjusting for age and sex, a higher proportion of participants in South Africa than Uganda reported being in good health (Fig. 2). For instance, nearly 60% of older people who were HIV infected and on ART in South Africa reported to be in good health compared to less than 40% in the same group in Uganda. Figure 2 suggests the differences in self-reported health were statistically significant for older people who were HIV infected (on treatment or not yet) and for those who had an adult child who was HIV infected. For the group of older people whose adult child had died of HIV-related causes, the differences between older people in South Africa and Uganda were not significant. As stated earlier, the comparison group (5) is only available in Uganda.

Similar results were observed in univariate and multivariable adjusted analyses. Here, in Table 3, we only present results from the multivariable analyses that compared the health status of study participants in South Africa to those from Uganda adjusting for sex, age group, education level, household wealth status, and rural–urban place of residence. The first three columns of Table 3 show the adjusted odds ratio (aOR) of being in good health by specific measure, whereas for the last two columns, it is the odds of being obese and reporting hypertension, respectively. In adjusted analyses, study participants in South Africa were about 28% less likely to be in good physical functional ability (WHODAS) relative to study participants from Uganda (Table 3). Among factors adjusted for sex, age group, and education were key determinants of the differences in the physical functional ability of participants in South Africa relative to Uganda.

**Table 3:** Multivariable regression results for selected health outcomes comparing Uganda to South Africa, 2010

	WHODAS^a^	WHOQoL^b^	BMI^c^	Self-reported hypertension	Self-rated health
	
	aOR (95% CI)	aOR (95% CI)	aOR (95% CI)	aOR (95% CI)	aOR (95% CI)
Uganda	1.00	1.00	1.00	1.00	1.00
South Africa	0.72 (0.53–0.98)	2.15 (1.60–2.90)	5.26 (3.46–8.00)	2.77 (2.06–3.73)	2.35 (1.74–3.17)
					
Sex					
Male	1.00	1.00	1.00	1.00	1.00
Female	1.54 (1.12–2.10)	1.78 (1.30–2.44)	1.17 (0.84–1.63)	1.37 (0.99–1.88)	1.84 (1.34–2.52)
					
Age group					
50–59	1.00	1.00	1.00	1.00	1.00
60–69	0.71 (0.51–0.99)	0.72 (0.51–1.01)	0.65 (0.45–0.92)	0.68 (0.48–0.96)	0.72 (0.51–1.00)
70–79	0.42 (0.28–0.61)	0.49 (0.33–0.72)	0.40 (0.26–0.60)	0.40 (0.27–0.59)	0.51 (0.35–0.75)
80 +	0.30 (0.17–0.52)	0.42 (0.24–0.73)	0.34 (0.19–0.62)	0.30 (0.17–0.53)	0.40 (0.23–0.69)
					
Education					
NFE/AEO^d^	1.00	1.00	1.00	1.00	1.00
Primary	0.29 (0.21–0.40)	0.26 (0.18–0.36)	0.25 (0.18–0.36)	0.28 (0.20–0.39)	0.27 (0.19–0.37)
Secondary	0.37 (0.23–0.60)	0.33 (0.20–0.53)	0.33 (0.20–0.55)	0.37 (0.23–0.61)	0.33 (0.20–0.54)
Tertiary	0.09 (0.03–0.28)	0.08 (0.03–0.24)	0.07 (0.02–0.23)	0.08 (0.03–0.26)	0.08 (0.03–0.26)
					
Wealth quintiles					
1 (lowest)	1.00	1.00	1.00	1.00	1.00
2	0.93 (0.60–1.44)	0.95 (0.61–1.45)	0.94 (0.59–1.50)	0.92 (0.59–1.44)	0.94 (0.60–1.47)
3	1.26 (0.81–1.96)	1.12 (0.72–1.75)	1.17 (0.73–1.86)	1.22 (0.78–1.91)	1.17 (0.75–1.83)
4	1.25 (0.81–1.95)	1.15 (0.73–1.80)	1.21 (0.75–1.93)	1.18 (0.75–1.85)	1.23 (0.78–1.92)
5 (highest)	1.37 (0.87–2.15)	1.30 (0.83–2.06)	1.12 (0.69–1.81)	1.38 (0.87–2.19)	1.36 (0.86–2.15)
					
Residency					
Peri-urban/urban	1.00	1.00	1.00	1.00	1.00
Rural	0.81 (0.61–1.07)	0.85 (0.64–1.12)	0.68 (0.50–0.92)	0.73 (0.55–0.98)	0.81 (0.61–1.08)

a WHO Disability Assessment Schedule (WHODAS) was used to assess functional ability.

b WHO Quality of Life (WHOQoL) instrument was used to assess subjective well-being.

c Body mass index, categorised into obese or not obese, using cut-off value of BMI ≥ 30.

d NFE/AEO – no formal education/adult education only.

In contrast, older people from South Africa were twice as likely as older Ugandans to report being in good subjective wellbeing and to rate their overall health as good. Despite their own perceived good health, South African study participants were more than five times (aOR 5.3, 95% CI 3.5–8.0) more likely to be obese (BMI ≥ 30) than their Ugandan peers. Likewise, South African older people had nearly three-fold increased odds of reporting hypertension relative to Ugandans (aOR 2.7, 95% CI 2.1–3.7). Age group, educational attainment, and rural/urban place of residence were significant factors in the increased odds of participants in South Africa relative to Uganda being obese and reporting hypertension (Table 3).

## Discussion

This study compared the health and wellbeing of older people in South Africa and Uganda based on a selected set of standardised health measurements. Study instruments used in the two sample surveys were mostly identical, and were adapted from the WHO–SAGE questionnaire. Some social and demographic characteristics differed between participants in South Africa and Uganda, with those in South Africa being slightly younger, more likely to be currently married, without formal education, not working, and residing in the more rural parts of the study area. For both sexes combined, older people in Uganda had slightly higher physical functional ability status, but lower subjective wellbeing status, than study participants from South Africa. Even after adjusting for several socio-demographic factors, participants in South Africa were statistically significantly less likely to have good physical functional ability than those in Uganda. Older people in South Africa had two-fold increased odds of being in good subjective wellbeing. However, South Africans were substantially more likely to have a BMI ≥ 30 or to be diagnosed with hypertension in adjusted regression analyses. Across all study groups, women were associated with higher BMI measures, except for Ugandan older men with the death of an adult child due to HIV causes. For this particular group, men had higher age–sex adjusted mean BMI measurements, which could have been influenced by one older man who had a BMI of 45.7 which may have been a measurement error probably in the height. This respondent was not dropped from the analysis.

The previous study in Uganda showed that ill-health such as having one of five listed chronic conditions, depression, hypertension, and poor vision were prevalent in Ugandan older persons (25), with women generally having poorer prognosis. Similarly, a study nested within another longitudinal surveillance in South Africa found older women were more likely to be in poor physical functional ability and overall health than their male peers (26). A major difference in the findings from these studies in Uganda and South Africa was that while in Uganda HIV-infected and HIV-uninfected older people had similar physical functional ability and health status, South African HIV-infected older persons had better physical functional ability, quality of life, and overall health than those who were HIV uninfected.

In this pooled analysis, similarities were found in socio-demographic factors like age, sex, education, and place of residency associated with the health and wellbeing status of older people in Uganda and South Africa. This is in spite of the setting and contextual factors of older people in the two countries being very different. For instance, while about four in five older people in Uganda were working, in South Africa only about one in ten respondents were currently working. Employment may be differently conceptualised in Uganda and South Africa, where in South Africa this variable referred almost exclusively to formal employment, while in Uganda it was more likely to refer to informal work as farm labourers or fruit and vegetable vending. Lack of comparability and reliability in this variable is the reason it was dropped from the multivariable regression analyses. According to data from the World Bank, the two countries have quite distinctly different development indicators (43). As of 2010, the population of South Africa was estimated at 50 million with an adult (15–49 years) HIV prevalence of 17.8%, while Uganda was estimated to have a population of 33 million and an adult HIV prevalence of 6.5%. The gross domestic product per capita in US dollars was 7,280 in South Africa and 509 in Uganda. Due to the higher HIV prevalence in South Africa, and despite a higher infant mortality rate in Uganda (63 per 1000 live births) than in South Africa (41 per 1000 births), life expectancy at birth is higher in Uganda (54 years) than South Africa (52 years) (43), which could partly be explained by high mortality due to HIV and accidents in early adulthood (44).

While a higher proportion of older people in Uganda may resort to working, usually on small pieces of land, in response to the lack of a social safety net in old age, in South Africa there is wide accessibility and uptake of non-contributory old-age pensions (45, 46). Other studies from South Africa have demonstrated that this old-age pension is an important and reliable source of income for the day-to-day household wellbeing, especially in rural South Africa (47). In populations severely affected by high HIV-related adult mortality, large government cash-transfers have been shown to help mitigate the consequences of older people caring for orphaned children (48). It has further been demonstrated that the risk of mental-ill health was lower among older people in receipt of government cash-transfer. It is thus not surprising that Ugandan respondents reported being in good physical functional ability but lower good subjective (emotional) wellbeing than those in South Africa, adjusting for age, gender, education attainment, occupational status, household wealth, and place of residency. The differences highlighted in this study may also be indicative of differences in health care accessibility, utilisation, or quality between South Africa and Uganda. There is evidence from elsewhere of a strong relationship between lower socio-economic status and poorer health and wellbeing in older people (17).

There were some limitations to this study. First, data were cross-sectional and no causal inferences can be drawn from the findings. Further, participants were selected *a priori* into study groups, thereby possibly introducing selection bias. However, as participants were selected from well-established cohort or population-based studies, the study samples were likely to be representative of older people in the respective study population. Second, there is a chance that some of the participants in the HIV-uninfected group could actually have been HIV infected. With the low HIV incidence in these settings, the widespread availability of HIV testing and access to treatment in both sites, this is unlikely to have been the case. Third, health status and wellbeing measures used in this analysis are largely based on self-reports, which may have limitations (36, 41). However, they have been shown to adequately measure health status in different socio-cultural and economic settings (36, 38). Finally, older people who participated in either site were based in the community; those who were institutionalised, hospitalised, or severely ill were excluded from either study, which could potentially have introduced a healthy selection bias. However, the consistent and distinct patterns in the health status by study country give confidence that these results are a fair representation of differences in the health and wellbeing of participants in Uganda and South Africa.

## Conclusion

While this study highlights similar health problems among older people in South Africa and Uganda, the socio-demographic differences in the studied populations influence the extent to which older people are affected by poorer health. These findings illustrate the importance of using a multifaceted approach to measuring the health status of older people and will be valuable inputs for local decision-making in each country. If, for instance, one were to only choose either BMI or self-reported hypertension to compare the health status of older people in South Africa and Uganda, one may conclude that the health status of participants in the latter is better than in the former. Using several measures of health status and adjusting for a number of socio-demographic characteristics, Ugandan respondents were more likely to be in good physical health but poorer emotional wellbeing than their South African counterparts. Improving the poorer health status of the Ugandan population requires not only investments in health care services, but also improvements in social services including consideration for introduction of some form of government cash-transfers, as is the case for older South African adults.

Results from this study suggest similar effects of HIV on the health and wellbeing of older people in the Ugandan and South African studies, with physical functional ability and emotional wellbeing health status highest among older people who were HIV infected and on ART, and lowest in participants with an adult child who had died with HIV (excluding the comparison group – older people who were not HIV infected or HIV affected, which could not be established in the South African sample). However, the exact impact of HIV infection and availability of HIV treatment on the physical health and functional ability of older people is more complex and may require robust population-based data to disentangle. There is therefore a need for further research.
